# Environmental Impact of Orthodontics: A Literature Review of Traditional Multibracket Appliances and Clear Aligners

**DOI:** 10.1155/ijod/2304712

**Published:** 2026-01-09

**Authors:** Antonino Peluso, Giovanna Murmura, Bruna Sinjari, Michele D’Attilio

**Affiliations:** ^1^ Department of Innovative Technologies in Medicine and Dentistry, University of Chieti-Pescara, Chieti, Italy, unich.it; ^2^ Department of Human Sciences, “Sustainable Blue Economy and One Health”—XL Cycle, Law and Economics “Leonardo da Vinci”, UNIDAV, Telematic University, Torrevecchia Teatina, Chieti, 66100, Italy; ^3^ High-Tech Dental Materials Laboratory, Department of Innovative Technologies in Medicine and Dentistry, University of Chieti-Pescara, Chieti, Italy, unich.it

## Abstract

**Introduction:**

Climate change has led to a growing interest in environmental sustainability in the healthcare sector, including orthodontics. This review aims to analyze and compare the environmental footprint of traditional multibracket appliances (TMAs) and clear aligners (CAs), focusing on five aspects: manufacturing process, pollution from raw materials, clinical management, and recycling potential.

**Materials and Methods:**

A comprehensive literature search was conducted on different databases: PubMed, Scopus, Web of Science, Google Scholar, and ScienceDirect. In addition, a manual and gray literature search was performed. Included studies were reviews, systematic reviews, epidemiological studies, or life cycle assessment (LCA) addressing environmental aspects of orthodontic materials and treatments. The articles included in the review were then divided into the following categories: manufacturing processes, environmental impact of production, clinical management, and generation of waste with recycling potential.

**Results:**

A total of 34 studies published between 2003 and 2004, along with academic books and technical/informational sources, were analyzed. The production of TMA materials relies mainly on stainless steel (SS) and alumina, while CA uses thermoplastic polymers such as polyethylene terephthalate (PET), glycol‐modified PET (PET‐G), and polyurethane (PU). Although SS production generates higher CO_2_ emissions, it offers better recyclability. In contrast, CA materials production requires more energy and generates a larger amount of nonrecyclable plastic waste. The environmental impact is also influenced by the clinical management of these therapies, such as the time and frequency of visits.

**Conclusion:**

Both systems contribute to environmental pollution. TMA appears more sustainable due to its recyclability and reduced waste generation. Future research should focus on improving recyclable polymers, sustainable production methods, and optimized clinical workflows.

## 1. Introduction

The climate crisis is the most significant challenge to human well‐being and planetary health that current and future generations will have to face [1]. Global average temperatures are significantly above preindustrial levels due to human activity and greenhouse gas (GHG) emissions [2].

Environmental sustainability has become a critical global issue, with increasing attention on how various industries contribute to pollution, waste generation, and carbon emissions. The global healthcare system, while essential for human well‐being, contributes to 4.4% of the total global environmental impact [3]. Orthodontic care, as part of this system, also contributes to environmental impact through the materials and resources consumed in treatments, from the manufacturing of orthodontic appliances to the frequency of patient visits and the disposal of materials.

Technological advances have led to the development of innovative orthodontic appliances, such as transparent aligners, which have broadened the range of treatment options available to patients. Consequently, an increasing number of individuals are seeking orthodontic care to correct malocclusion and improve dental esthetics.

The choice between traditional multibracket appliances (TMAs) and clear aligners (CAs) not only influences the therapeutic experience of the patient but also has a substantial environmental impact.

TMA uses conventional brackets and wires that require periodic adjustments during treatment, whereas CA consists of a series of removable thermoplastic devices, custom‐fitted to the patient’s teeth, that must be periodically replaced.

Given the growing focus on sustainability, it is important to assess whether CA, often promoted as more convenient and esthetically pleasing, is also more environmentally sustainable compared to TMA.

To the authors’ knowledge, no previous study has comprehensively evaluated the environmental impact of these two treatment modalities. Therefore, this review aims to compare the environmental footprint of CA and TMA across the main stages of their life cycle—from production to clinical use and end‐of‐life management.

## 2. Materials and Methods

This review aims to assess and compare the potential environmental impact of CAT and TMA. In order to achieve the review scope, comprehensive literature research was conducted across several databases (PubMed, Scopus, Web of Science, Google Scholar, and ScienceDirect) from November 2024 to March 2025.

To further enhance the literature research, a manual search was carried out through the reference lists of the identified articles, and in addition, a gray literature search was performed. Data and information from the gray literature were considered only if they originated from authoritative sources, such as government reports or recognized scientific societies.

The inclusion criteria were the following:

– Reviews and systematic review.
– Epidemiological studies.
– Life cycle assessment (LCA) analysis.

The exclusion criteria were the following:

– Articles published in a non‐English language.
– Opinion articles.
– Case series.
– Case reports.

The suitable items were independently evaluated by Antonino Peluso, and in case of uncertainties, a second author (Michele D’Attilio) was consulted.

The selected studies were organized into the following distinctive sections based on their focus areas:

– Materials and orthodontic appliance manufacturing: This section examined the materials used in the production of orthodontic devices and the process by which the orthodontic devices are manufactured.
– Manufacturing environmental impact: This section analyzed the environmental effects associated with the production of orthodontic materials needed for the realization of orthodontic devices.
– Frequency of visit and emergencies: This category assessed the environmental impact associated with the transportation required for patient visits, taking into consideration the frequency of scheduled and emergency appointments.
– Waste generation and recycling: This section explored the types and amounts of waste generated during orthodontic treatments, as well as the potential for recycling.

The terms used in conducting the research are presented in Table 1, categorized for each specific item.

**Table 1:** Research terms.

Item	Search terms
Materials and orthodontic appliance manufacturing processes	Orthodontic ^∗^ AND (bracket ^∗^ OR aligner) AND (material OR production OR manufac ^∗^) NOT (biomechanics OR biocompatibility)

Manufacturing environmental impact	– (stainless‐steel OR alumina) AND (“life cycle assessment” OR pollution OR “environment ^∗^ impact”) – (polyurethane AND PET‐G) AND (“life cycle assessment” OR pollution OR “environmental impact”) – (orthodontic ^∗^ OR aligner ^∗^) AND (pollution OR environment ^∗^) NOT (microbio ^∗^ or bisphenol)

Frequency of visit and emergencies	(aligners OR “fixed appliances”) AND (“treatment effectiveness” OR “treatment outcomes”)

Waste generation and recycling	(Orthodontic ^∗^ OR aligner ^∗^ OR bracket ^∗^) AND (waste OR recycling OR recondition ^∗^ OR reuse)

## 3. Results

A total of 34 references, including studies, academic books, and technical/informational sources published between 2003 and 2024, were analyzed in this narrative review.

The following sections present a qualitative synthesis of the collected evidence, highlighting the main findings within each thematic area.

### 3.1. Materials and Orthodontic Appliance Manufacturing Processes

#### 3.1.1. Multibrackets Appliance

Over the years, orthodontic appliances have undergone significant evolution. This has led to the development of new high‐performance materials with the aim of improving esthetics and clinical performance.

Brackets can be manufactured from various types of materials, including metals, ceramics, plastics, and others. However, the most commonly used types are stainless steel (SS) and alumina‐based ceramic brackets [4]. To achieve the desired tooth movement, these brackets are connected to a nickel‐titanium or SS wire by means of elastic modules if self‐ligating brackets are not used.

In the course of TMA orthodontic treatment, several auxiliary components are utilized, such as intermaxillary elastics, power chains, metal ligatures, and coil springs.

The SS alloys most used in the production of orthodontic brackets are 303, 304, and 316 L. However, due to concerns over nickel allergies, alternative SS types, such as the 2205 duplex alloy, have been introduced.

Currently, there are two main methods employed for manufacturing SS orthodontic brackets: metal injection molding (MIM) and 3D printing. Among these, MIM is the most used technique. This technique mixes fine metal powder with binders and then injects the mixture into a mold to produce a one‐piece bracket. Postproduction, greater accuracy can be achieved by milling the bracket slot [4, 5].

Ceramic brackets available nowadays are primarily made from aluminum oxide (alumina), which can be either polycrystalline or monocrystalline. Monocrystalline alumina is significantly stronger than polycrystalline alumina; however, both types exhibit poor fracture toughness [6].

Polycrystalline brackets are commonly produced using the ceramic injection molding (CIM) method. In this process, aluminum oxide particles are mixed with a binder to form a mixture, which is then injected into bracket molds. After removing the binder, sintering is carried out to densify the material. The advantage of CIM is the possibility to mass–produce complex, precise components with smooth surfaces, in large quantities at a high rate.

The manufacturing process for monocrystalline alumina brackets is different. The alumina particles are melted and slowly cooled to facilitate controlled crystallization, forming a large single crystal. This crystal is then milled into brackets with ultrasonic cutting techniques and/or diamond cutting tools. After milling, the monocrystalline brackets undergo heat treatment to eliminate surface imperfections and relieve stress induced by the milling procedure [7].

All the results are summarized in Table 2.

**Table 2:** Materials used and manufacturing process for each type of bracket.

Bracket type	Material	Manufacturing process
Metallic bracket – Srindhi et al. [4]	– Stainless steel 303 – Stainless steel 304 – Stainless steel 316 – Stainless steel 2205 duplex alloy	– Metal injection molding (MIM) – 3D printing

Ceramic bracket – Mundhada et al. [6] – Elekdag‐Türk and Yilmaz [7]	– Polycrystalline alumina – Monocrystalline alumina	– Ceramic injection molding – Ultrasonic milling or diamond cutting

#### 3.1.2. CA

In recent years, the number of patients seeking orthodontic treatment has significantly increased. This rise in demand is accompanied by a growing preference for more esthetic appliances, with CAT gaining considerable attention.

The most used workflow for creating orthodontic aligners begins with the virtual treatment planning, which necessitates obtaining an impression of the patient’s dental arches. The impression can be obtained using traditional impression material or by means of an intraoral scan. Subsequently, a physical 3D model is printed for each individual aligner, and a clear thermoplastic material is molded over the physical models [8].

Three‐dimensional printing has enabled the possibility to print the aligners directly, eliminating the model production and the thermoforming (THF) steps. This process, also called additive manufacturing, is based on adding material layer upon layer with different methods (material extrusion, material jetting, powder bed fusion, vat photo‐polymerization, etc.) [9, 10]. However, 3D printing by photo‐polymerization from clear resin is currently the most suitable option [11].

The most used materials to produce CAs are polyurethane (PU), polyethylene terephthalate (PET), and glycol‐modified PET (PET‐G) [12].

CA materials have undergone a significant transition from single‐layered or monophase plastics to the third‐generation multilayered materials.

These modern materials typically consist of both hard and soft layers. The soft layer facilitates elastic deformation, which promotes the aligners’ seating, while the hard layer provides strength and durability [13].

PET combines ethylene glycol (EG) with terephthalic acid (TPA) and exists in both amorphous and crystalline forms [14]. The amorphous structure is transparent and exhibits excellent ductility, while the crystalline structure is opaque and white and exhibits hardness, stiffness, and good strength [15]. PET can be rigid or semi‐rigid depending upon the processing methods employed and displays excellent mechanical properties, toughness, and solvent resistance [16].

PET‐G is a non‐crystalline co‐polyester, comprised of 1,4‐cyclohexane, two methanol (CHDM), EG, and TPA [17].

PETG shows excellent transparency, resistance against various solvents, and can either be printed or thermoformed [18].

PU is a highly versatile polymer known for its excellent mechanical and elastomeric properties. When exposed to stress, PU deforms under load but can return to its original shape once the load is removed, demonstrating significant elongation and recovery due to the material’s inherent flexibility [12].

All the results are summarized in Table 3.

**Table 3:** Material and production process in CAT.

Material	Manufacturing process	Study
– Polyurethane (PU) – Polyethylene terephthalate (PET) – Glycol‐modified polyethylene terephthalate (PET‐G)	– Thermoforming – 3D printing	– Tartaglia et al. [8] – Maspero and Tartaglia [11] – Bichu et al. [12]

### 3.2. Manufacturing Environmental Impact

To assess the environmental impact of various production methods, an understanding of the materials’ manufacturing process is essential. Due to the limited availability of articles specific to orthodontic and dentistry manufacturing, insight derived from the material science literature must be considered.

This review considered life cycle analysis (LCA) focusing on the “cradle‐to‐gate” phase—from raw materials acquisition to the factory gate, excluding the product’s use and end‐of‐life [19].

#### 3.2.1. SS

SS is produced through a two‐stage process. Initially, raw materials are melted together in an electric arc furnace. The molten metal is then transferred to an argon‐oxygen decarburization (AOD) vessel, which lowers impurities, reducing the carbon content to the low levels required for the final product [20].

Norgate et al. [20, 21] have evaluated various aspects of the SS life cycle, focusing on its environmental impact through metrics such as

– Gross energy required (GER), representing the total amount of primary energy consumed.
– Global warming potential (GWP), indicating GHG associated with production.
– Acidification potential (AP), measuring sulfur dioxide emission.
– Solid waste burden (SWB) reflecting the amount of solid waste generated during production.

According to their results, the production of 1 ton of SS generates 75,000 MJ of GER, 6800 kg CO_2_e of GWP, 51 kg SO_2_e of AP, and 6400 kg of SWB.

Norgate et al. [20] also examined the environmental impact of SS produced using different nickel sources: ferronickel and nickel metal. Assessing this aspect is particularly relevant to orthodontic brackets, which are made from SS type 303, 304, and 316 L. In particular, SS 303 and 304 are produced using ferronickel, while SS 316 L uses nickel metal as a nickel source.

The GER needed for producing 1 ton of material from ferronickel is 75,000 MJ, compared to 49,000 MJ for nickel metal.

The GWP from ferronickel is 6800 kg CO_2_e and 4900 kg CO_2_e from nickel metal. AP is 51 kg SO_2_e for ferronickel and 38 kg SO_2_e for nickel metal.

Jhonson et al. [22] analyzed the environmental impact of SS production under three scenarios: current operation (modern‐day production methods), maximum recycling (an ideal condition where the SS is 100% recycled), and virgin production (no recycled content).

Taking into consideration only the LCA for the current operation scenario, their finding estimates that to produce 1 ton of SS, the energy consumption is 53,000 MJ, with the melting process requiring the most amount of energy. Transportation contributes 7%–8% and the extraction and transport of minerals contribute less than 3%.

Fossil fuels provide 81%–88% of the total energy use, and the fuel production and transportation contribute ~10%. CO_2_ emission for the current operation method is 3600 kg CO_2_ per ton of SS.

#### 3.2.2. Alumina

Alumina (Al_2_O_3_) is another important material in orthodontics for the manufacturing of ceramic brackets. The primary ore of relevance for its manufacturing is the bauxite, a mixture of aluminum hydroxides and oxyhydroxides with varying amounts of iron oxides, silicates, and other impurities [23]. Alumina is obtained from bauxite through the Bayer process, patented by Karl Bayer in 1887 [24]. Despite its longevity, it continues to be the most important process used to produce alumina on an industrial scale, and it has been optimized throughout the years [25].

Saez‐Guinoa et al. [26] have published an in‐depth analysis of the environmental impact associated with the Bayer process used for alumina production. According to their results, the GER to produce 1 ton of alumina accounts for 8554 MJ, while the GWP is 988 kgCO_2_e when natural gas is used as a reference fuel. Natural gas combustion contributes to 65% of the total GHG during alumina production, and other major contributors include Bauxite transportation (13%) and sodium hydroxide (NaOH) usage (11%). The remaining contributors (electricity use and solid waste) account for less than 10% of total emissions. Regarding the AP, its values are of 3.4 kg SO_2_e, with the Bauxite maritime transport as a major contributor, representing 64% of total emission due to the release of sulfur and nitrogen.

Another recent study by Ma et al. [27] focuses on LCA of alumina manufacturing. According to their findings, the GER for manufacturing 1 ton of alumina is ~10,371 MJ, while, in terms of environmental impact, the study reports that the GWP accounts for 1648kgCO_2_e. Furthermore, the study assessed that the AP value is estimated at 7.15 kg SO_2_e and the SWB is about 1590 kg per ton of alumina.

#### 3.2.3. PET, PET‐G, and PU

Regarding the environmental impact of plastics used in aligners, the results concerning PET are derived from the review by Walker and Rothman [28]. This manuscript considers studies that evaluate the environmental impact of fossil‐based plastics to subsequently compare them with bio‐based plastics. For the purposes of this review, the values of fossil‐based PET were taken into consideration. Although both cradle‐to‐gate and cradle‐to‐grave LCAs were evaluated, only the cradle‐to‐gate results have been considered for the objectives of this review.

The findings from this study indicate that the production of 1 ton of PET has an environmental impact in terms of GER, amounting to 80,000 MJ. In terms of AP, the reported values range from 10 to 20 kg SO_2_e, while the GWP is quantified at 5000 kg CO_2_e. However, the authors point out that their review reveals considerable variations in the findings reported. According to the authors, this inconsistency is attributed to different methodologies used in the analysis of existing literature; thus, further studies are needed to achieve more accurate results.

Regarding thermoplastic PU, no articles deemed suitable for the objectives of this review were identified in the literature.

**Figure 1:**
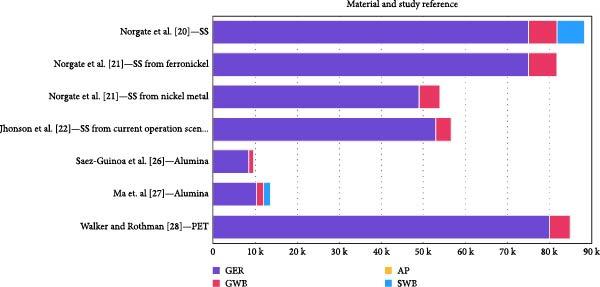
Chart illustrating the environmental impact of the material used in orthodontic appliance manufacturing.

All results are shown in Table 4 and illustrated in Figure 1

**Table 4:** Data from studies on the manufacturing processes of each material.

Study	Material	Manufacturing process	GER (MJ)	GWP (kg CO_2_e)	AP (kg SO_2_e)	SWB (kg)
Norgate et al. [20]	SS	Electric arc furnace + AOD	75,000	6800	51	6400
Norgate et al. [21]	SS from ferronickel		75,000	6800	51	‐
	SS from nickel metal		49,000	4900	39	‐
Jhonson et al. [22]	SS from current operation scenario		53,000	3600	_	‐

Saez‐Guinoa et al. [26]	Alumina	Bayer process	8554	988	3.4	‐
Ma et. al [27]			10,371	1648	7.15	1590

Walker and Rothman [28]	PET	Polycondensation	80,000	5000	10–20	‐

*Note:* Results referred to 1 ton of material.

To date, despite the limited availability of articles related to orthodontic manufacturing, only one study from Caelli et al. [29] has compared two methods of aligner production: THF and direct printing (DP) with digital light processing (DLP) technology.

The analyzed system includes the production from raw materials to aligners’ manufacturing.

Due to the unavailability of data, it was impossible for the authors to include aligners use and disposal.

The functional unit taken into consideration is a complete set of 40 aligners.

The results showed that DP generally produces a lower environmental impact compared to THF.

In particular, GER decreases from 188.9 MJ eq for THF to 52.15 MJ eq for DP.

**Figure 2:**
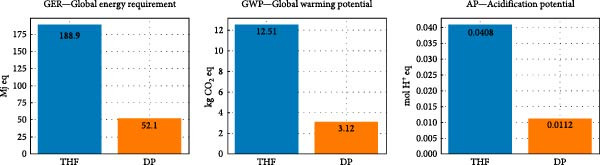
Environmental impact comparison between THF and DP.

The value of GWP is of 12.51 kg CO_2_ eq for THF and 3.12 kg CO_2_ for DP, while AP reduced from 0.0408 mol H^+^eq for THF to 0.0112 mol H^+^eq for DP (Figure 2).

This reduction is mainly due to the decreases in material and energy use.

Although the SWB is not explicitly evaluated in the study, the authors report a reduction of 70%–75% in waste generation for DP, due to the elimination of resin model and lower material and water consumption.

These findings suggest that DP can reduce the environmental footprint of aligner manufacturing thanks to improved material efficiency and energy saving.

### 3.3. Treatment Time, Visit Frequency, and Emergencies

The environmental impact of orthodontics does not only rely on the material used for appliance manufacturing, but also on the clinical management employed throughout the treatment process. Among the critical aspects of orthodontic care, treatment duration, follow‐up frequency, and the handling of emergencies play a decisive role in the environmental impact.

According to the current literature, there is no homogeneous consensus on the treatment efficiency between CAs and fixed appliances.

Different systematic reviews have tried to assess the treatment time between these two techniques, in particular, two systematic reviews by Jaber et al. [30] and Alhamwi et al. [31] conclude that CAs are more time efficient in the treatment of mild to moderate malocclusion compared to fixed conventional appliances, but it takes a longer time in the treatment of complex malocclusion. Additionally, a systematic review by Papageorgiou et al. [32] pointed out that patients treated with aligners are more likely to finish with unacceptable quality compared to the American Board of Orthodontic (ABO) standard, and for this reason, CATs are more likely to need further refinement, which leads to longer treatment time.

According to the aforementioned studies, all agreed that treatment with TMA needs more chair time and more emergency visits, but findings from Alhamwi et al. [31] highlight that CAT needs a higher overall number of appointments.

Understand this aspect is of fundamental importance for an in‐depth analysis of the environmental footprint of orthodontic therapy, as every dental examination has a certain environmental impact, as demonstrated by a study from Borglin et al. [33].

Broglin et al. [33] have quantified the environmental burden of a dental examination using the LCA technique for 16 impact categories. In particular, the functional unit taken into consideration included a 15‐min examination performed by a dentist and a dental nurse.

As X‐ray procedures were not included in the functional unit, the study provides a representative analysis of a conventional orthodontic appointment, whether scheduled or unscheduled.

The main findings indicate that each dental examination produces ~0.73 kg of CO_2_e emissions, equivalent to driving 4.55 km in a small car. The most significant environmental impacts are found in categories such as water scarcity (0.43 m^3^ deprivation), freshwater eutrophication (1.71E‐04 kg Pe), human toxicity and cancer effects (1.19E‐08 CTUh).

Key contributors to the environmental burden include:

– Soaps and detergents significantly impact nearly all categories except ionizing radiation and photochemical ozone formation.
– Disposable bibs contribute to 11 out of 16 impact categories, including freshwater eutrophication and human toxicity (cancer effects).
– Surface disinfectants, responsible for 90% of the potential photochemical ozone formation and are highly impactful in nine categories, including acidification and noncancer human toxicity.
– SS instruments, which are the largest contributors to cancer‐related human toxicity and are also significant in mineral and metal resource use, freshwater eutrophication, and respiratory effects.

As demonstrated by Broglin et al. [33], disposable products are the primary contributors to pollution in orthodontic care. Although CAT orthodontic visits require less time than those for TMA therapies, this does not reduce their environmental impact, as most pollution stems from single‐use items. In fact, due to the shorter duration of aligner visits, allowing more appointments per day, these visits may increase their cumulative environmental impact.

Another important aspect to take into consideration is the environmental impact deriving from patients’ transportation. To date, the transport‐derived pollution is quantified in a study conducted by Duane et al. [34].

The geographical area of reference is the United Kingdom, and the study states that dentistry, unlike other services of the National Health System, has a significant amount of its carbon emissions coming from transportation. In particular, 64.5% of the carbon emissions related to dentistry are due to transportation, of which 31.1% is attributable to patient transportation.

This study estimated that the average distance traveled by patients for a dental appointment is 7.57 miles (12.18 km) for an annual total of 480 miles traveled by patients. This determines an annual release of 224.09 tons of NOx and 11.01 tons of PM 2.5 (respectively 3.58 × 10^−6^ tons of NOx and 1.75 × 10^−7^ tons of PM 2.5 for 7.57 miles). All the studies are summarized in Table 5

**Table 5:** Summary of studies on treatment duration and pollution.

Study	Focus	Study type	Conclusion
Jaber et al. [30]	Treatment time of CA vs TMA	Systematic review	CAs are more efficient in mild/moderate malocclusion; in complex cases treatment time is longer compared to TMA

Alhamwi et al. [31]	Treatment time of CA vs TMA	Systematic review	CAs are more efficient in mild/moderate malocclusion; in complex cases treatment time is longer compared to TMA

Papageorgiou et al. [32]	Efficacy of CA and TMA	Systematic review and meta‐analysis	Patient treated with CA often fail to reach ABO standard, so further refinement are required. This led to longer treatment duration and an increased number of aligner

Broglin et al. [33]	Environmental burden of a dental visit	Life cycle assessment (LCA)	Each visit produces 0.73 kg of CO_2_e emission

Duane et al. [34]	Patient travel and carbon emission	Observational study	64.5% of dental sector emissions are due to transportation; 31.1% attributable to patients; 12.18 km is the average distance per visit.

**Figure 3:**
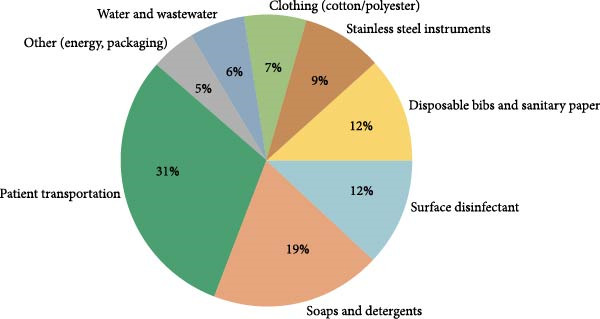
Combined environmental impact of orthodontic care.

Figure 3 illustrates the integrated contribution of clinical and transport‐related environmental impact of orthodontics from Broglin et al. [33] and Duane et al. [34].

### 3.4. Waste Generation and Recycling

The environmental impact of waste management and recycling in orthodontic therapies involves evaluating the materials used in both therapies.

Waste materials generated from metallic brackets and orthodontic wires include SS alloys and nickel‐titanium alloys. While these materials are recyclable, specific challenges limit their recycling in orthodontics. These include contamination with biological material and residual adhesive or composite.

Juman et al. [35] have analyzed with LCA methodology the amount of waste generated by an orthodontic practice.

Their study has taken into consideration only the waste generated from TMA and has estimated that 97% of the total waste is incinerated and 3% is landfilled.

A total of 32% of the waste generated comes from conventional impression taking, 24% from cross‐infection control and disinfection, and 20% from personal protective equipment. Other minor sources of waste are disposable items with reusable alternatives (6%), contaminated sharp (1%), and paper towels (3%).

To allow safe insertion, all the arch wires are trimmed extra orally. The authors have also quantified the amount of waste (excess arch wire length) generated in the different stages of orthodontic treatment.

Initial aligning arch wire is 38% in excess, while for the sequence arch wire, the amount of excess is 26% and for the working arch wire, the amount is 39%.

Used brackets (either metal or alumina) can currently be reconditioned for use on other patients, enabling their application in new treatments. However, this practice is restricted to specialized companies that operate bracket return systems [36].

Conversely, ligatures and power chains are single‐use, nonrecyclable components and are thus destined for incineration.

CAT instead used plastic such as PET, PET‐G, and PU that are technically recyclable, but several challenges prevent their recycling. Aligners are composed of multiple layers of a different plastic, which means that they cannot be handled through conventional household recycling procedures.

Currently, different companies provide a collection system for used aligners. However, these aligners are then destined to produce other materials through secondary recycling [37] or utilized as fuel for heat or electricity generation through quaternary recycling [38].

In conclusion, the literature lacks specific studies aiming to quantify the waste management of orthodontic and specifically for CAT and TMA.

## 4. Discussion

In recent years, there has been growing attention to the environmental impact of the orthodontic treatments, especially with the increase in aligner therapies.

This review aims to provide an in‐depth comparison of the environmental impact of the two treatment modalities. The results of this review have highlighted several critical issues related to the production process and life cycle of these types of appliances.

SS and alumina require substantial energy for manufacturing but are recyclable and can be reintegrated into production. High temperature and significant energy consumption are needed to produce SS. Similarly, alumina necessitates complex extraction and purification processes, but once produced, it is chemically stable and has a long service life, a characteristic that mitigates its ecological burden.

In contrast, CA uses thermoplastic polymers that come from fossil fuels. Their manufacturing contributes to GHG and resource depletion. The use of multilayered polymers makes recycling technically difficult, and the THF process produces a significant amount of plastic waste, which worsens the environmental impact.

The ecological footprint is also influenced by the clinical management. Fixed appliances typically involve longer appointments and occasional emergencies, whereas aligner treatments, despite shorter visits, generally require a higher total number of appointments.

Every visit necessitates the use of energy, disposable materials, and patient transportation, all of which contribute to indirect emissions. Although TMA appointments are typically longer, their environmental burden per visit does not increase proportionally with chair time. The main contributors–single‐use item, cleaning agent, and patient travel–are relatively constant regardless of appointment length [33]. Consequently, while TMA visits last longer, the cumulative footprint of CA treatments tends to be higher overall because of the greater number of visits required.

Therefore, reducing visit frequency through optimized treatment planning and adopting digital monitoring technologies can effectively mitigate these impacts for both modalities.

Waste management is still a big issue. Although brackets and metallic components can be reconditioned by specialized facilities, aligners cannot be processed through conventional recycling, and they are usually incinerated or converted into secondary materials. These limitations emphasize the need for further innovation in sustainable polymers and in improving the recycling pathways for orthodontic plastics.

A limitation of this review lies in the quantitative findings derived from LCA of raw materials rather than from orthodontic‐specific studies. This reduces the possibility of drawing absolute numerical comparisons and highlights the necessity of future LCAs specific to orthodontic devices and workflows.

In general, neither treatment approach can be considered entirely sustainable. However, TMA currently seems to have a lower environmental burden when considering recyclability, material efficiency, and waste management. This is mainly because of their recyclable metallic components and decreased plastic waste. CA, despite their benefits for patient comfort and appearance, CA increase indirect emissions and plastic waste.

Therefore, clinicians should aim to reduce the ecological footprint of orthodontic care through optimized treatment duration, fewer in‐person visits, and a more responsible use of disposable materials. Supporting research on recyclable polymers and sustainable production methods will be essential to advancing toward a more environmentally friendly orthodontic practice.

## 5. Conclusion

This review is the first to comprehensively compare the environmental impact of TMA and CA. The results show that both types of treatment present environmental issues: TMA materials require high energy for manufacturing but are more recyclable, whereas CA generates more plastic waste and requires more appointments.

Clinicians can help lower the ecological footprint of orthodontic care by accurate treatment planning in order to reduce visit frequency and treatment duration. In addition, digital dental monitoring, recyclable materials, and the minimum use of disposable items can further enhance sustainability.

Further studies specific to orthodontics are needed to better understand the environmental footprint of the two appliances and reduce their impact without compromising clinical efficacy.

Based on this review, several considerations should be made when choosing the most sustainable treatment: type of orthodontic device, treatment time, material consumption, number of visits, and patient travel distance.

## Conflicts of Interest

The authors declare no conflicts of interest.

## Funding

This research received no external funding.

## Data Availability

The data that support the findings of this study are available from the corresponding author upon reasonable request.
